# Endogenous Circadian Clock Machinery in Cortical NG2-Glia Regulates Cellular Proliferation

**DOI:** 10.1523/ENEURO.0110-22.2022

**Published:** 2022-10-04

**Authors:** Terry Dean, Aissia Victoria Koffi, Evan Goldstein, Javid Ghaemmaghami, Vittorio Gallo

**Affiliations:** 1 Center for Neuroscience Research, Children’s National Hospital, Washington, DC 20010; 2 Howard University, Washington, DC 20059; 3 Division of Critical Care Medicine, Children’s National Hospital, Washington, DC 20010

**Keywords:** Bmal1, circadian, NG2-glia, OPC

## Abstract

The molecular circadian clock can be found throughout the body and is essential for the synchronizing cellular physiology with the 24 h day. However, the role of the clock in regulating the regenerative potential of the brain has not been explored. We report here that murine NG2-glia, the largest population of proliferative cells in the mature central nervous system, rhythmically express circadian clock genes in a 24 h period, including the critical clock component *Bmal1* RNA and BMAL1 protein. Interestingly, daily NG2-glia proliferation preferentially occurs during the time of day in which *Bmal1* expression is high, while conditional knockout of *Bmal1* decreases both cortical NG2-glia density and cellular proliferation. Furthermore, in a neurotrauma model, we show that pathology-induced NG2-glia proliferation is also dependent on *Bmal1* expression. Because circadian rhythm disturbances are common in neurologic disorders across the life span, including in traumatic brain injury, these findings bear significant implications for cellular regeneration in brain injuries and disease.

## Significance Statement

Circadian rhythm disturbances are commonly seen in neurologic disorders across the life span. The ramifications of these disturbances for the cellular healing capabilities of the brain are unknown. We show here that the largest population of regenerative cells in the adult central nervous system, known as NG2-glia, are indeed dependent on the integrity of their endogenous circadian rhythms. They not only rhythmically express molecular clock components, but the critical clock gene *Bmal1* plays an important role in regulating their ability to proliferate, both at rest and after injury. These findings underscore the importance of circadian dysregulation in affecting brain recovery at the cellular level in neurologic disease.

## Introduction

Cells throughout the body are regulated at the molecular level by the circadian clock. The rhythmic expression of transcriptional activators and inhibitors that comprise the molecular clock synchronizes cellular physiology with the 24 h day (Takahashi, 2017). Thus, several of the tissues and functions of the body are governed by circadian rhythms (Patke et al., 2020). However, the impact of circadian rhythms on the regenerative potential of the brain has not yet been explored. This holds significant implications for recovery from CNS injuries and disease, as many neurologic disorders throughout the life span cause significant circadian disturbances themselves (Logan and McClung, 2019).

NG2-glia, also called oligodendrocyte precursor cells or polydendrocytes, are a relatively recently discovered glial cell type that comprise the largest population of regenerative cells in the mature CNS (Dimou and Gallo, 2015). Found throughout the adult brain, they are most commonly characterized as ramified cells expressing oligodendrocyte markers (e.g., Olig2, Sox10) in addition to two proteins not expressed in postmitotic oligodendrocytes: NG2 and PDGFRα. They are capable of cellular proliferation, both at a basal rate and in response to injury (Hughes et al., 2013; Adams and Gallo, 2018). Additionally, they are motile, surveil the local environment with their cellular processes, participate in direct cell–cell signaling, and can differentiate into mature oligodendrocytes (Hughes et al., 2013; Li et al., 2020). However, it is unknown whether any NG2-glia functions such as these are beholden to the circadian clock. While sleep–wake state may influence NG2-glia proliferation (Bellesi, 2015), the impact of circadian timekeeping, itself, has not been rigorously explored (Colwell and Ghiani, 2020). Furthermore, the cell autonomy of a circadian influence on NG2-glia functions is unknown, as NG2-glia do receive direct neuronal inputs and have not yet been shown to endogenously express clock genes.

We present here a line of investigation revealing that NG2-glia indeed possess circadian clock components, and that expression of clock genes oscillates with a 24 h period. Furthermore, we show that the circadian clock and *Bmal1* expression, considered a principal driver of the mammalian circadian clock, regulates cortical NG2-glia proliferation and density in the naive, uninjured brain, as well as in response to injury.

## Materials and Methods

### Animals

Colonies of the following mice were maintained at the Children’s National Hospital Research Animal Facility: C57BL/6 mice [catalog #003548, The Jackson Laboratory (Jax)]; B6N.Cg-Tg(*Pdgfra-cre/ERT*)467Dbe/J (“*Pdgfra*-creERT”; catalog #018280, Jax) transgenic mice; B6.129 × 1-*Gt(ROSA)26Sor^tm1(EYFP)Cos^*/J (“R26R-EYFP”; catalog #006148, Jax) transgenic mice; B6.129S4(Cg)-*Arntl^tm1Weit^*/J (“*Bmal1*(fl/fl)”; catalog #007668; Jax) transgenic mice; and B6;FVB-Tg(*Pdgfra-EGFP/Rpl10a*)JD340Htz/J (“*Pdgfra*-TRAP,” catalog #030268, Jax) transgenic mice. Animals were maintained in a C57BL/6 genetic background, and breeding crosses were set to yield the final genotypes and corresponding controls (Ctls) required for the following experiments, as described. Male and female mice were used for experiments and were initially housed in the general colony in groups of two to five mice per cage under standard vivarium conditions with a 12 h light/dark cycle and *ad libitum* access to standard laboratory chow and water. For experiments in which circadian time differences were to be measured (e.g., circadian clock gene RNA or protein expression, NG2-glia cell density), male mice were transferred to a light-tight, sound-isolated circadian cabinet for a minimum of 2 weeks for entrainment in a 12 h light/dark cycle, followed by 24 h of constant darkness before collection at the designated circadian times. Animals undergoing translating ribosome affinity purification (TRAP) isolation of RNA underwent dissection and immediately proceeded to homogenization, as described below. Animals undergoing RNA *in situ* hybridization (ISH) and immunofluorescence staining underwent perfusion with PBS and 4% paraformaldehyde (PFA), followed by serial sucrose dehydration and freezing in optimal cutting temperature (O.C.T.) compound at −80°C. All animal procedures were performed ethically, and all protocols were approved by the Institutional Animal Care and Use Committee and in accordance with the *Guide for the Care and Use of laboratory Animals* (National Institutes of Health) and the *Policies on the Use of Animals and Humans in Neuroscience Research* (Society for Neuroscience).

### Conditional knockout

Conditional knockout (CKO) was achieved through the use of the tamoxifen-inducible Cre-loxP system restricted to *Pdgfra*-expressing cells, as previously described (Adams et al., 2020). Tamoxifen (Sigma-Aldrich) was dissolved in 100% ethanol and then diluted in sunflower oil (Sigma-Aldrich) to a final concentration of 10 mg/ml. *Pdgfra*-creERT:*Bmal1*(fl/fl) and *Bmal1*(+/+) controls were injected intraperitoneally with 75 mg/kg tamoxifen once per day for 5 d starting at postnatal day 28 (P28) or P36, depending on the experiment; the main text provides the timelines for the CKO experiments. One additional cohort of animals underwent sleep recording via a previously validated, noninvasive, piezoelectric sleep detection system (Signal Solutions; Mang et al., 2014; Yaghouby et al., 2016). These mice were individually housed within a light-tight, sound-isolated circadian cabinet for 10 d before tamoxifen injection. Following tamoxifen injection, the mice were maintained in the sleep recording apparatus for 1 week. Sleep during the light and dark phases were compared before and following tamoxifen injections.

### “Hit and run” model of TBI

We adapted the “Hit and Run” model of traumatic brain injury (TBI; Ren et al., 2013) to be able to provide reliable, closed-head injuries in an expedient manner necessary for experiments sensitive to time of day. Anesthetic induction was provided via ketamine (80 mg/kg, i.p.) and xylazine (8 mg/kg, i.p.). After appropriate nonresponsiveness was achieved, mice were moved to a water recirculating heating pad at 37°C. For each TBI and sham procedure, mice were shaved, and marked at a point halfway between the right eye and right ear. Each mouse was hung from its incisors and moved into position for an electromagnetic piston (Impact One, Leica) to provide an impact normal to the indicated mark with the following settings: speed, 5.2 m/s; depth, 10 mm; contact time, 0.1 s. All mice were provided with an analgesic dose of buprenorphine (0.05 mg/kg, i.p.) and recovered in a warmed cage (37°C) until the resumption of normal behaviors when it could rejoin its littermates. For CKO experiments, *Pdgfra*-creERT:*Bmal1*(fl/fl) animals injected with tamoxifen were compared with pooled controls (tamoxifen-injected and vehicle-injected animals).

### Translating ribosome affinity purification

RNA was isolated from *Pdgfra*-TRAP mice for RNA sequencing and for specific quantitative PCR (qPCR) evaluation of circadian genes. Briefly, subcortical white matter or whole brains, respectively, were rapidly dissected in ice-cold dissection buffer, homogenized in lysis buffer containing dithiothreitol (DTT), cycloheximide, rRNasin, Superasin, and EDTA-free protease inhibitors, and then centrifuged at 1000 × *g* for 10 min. After 1% NP-40 and 1% DHPC (1,2-dihexanoyl-sn-glycero-3-phosphocholine) were added to supernatants, they were chilled on ice for 5 min and centrifuged at 10,000 × *g* for 10 min. Supernatants were incubated overnight at 4°C with GFP antibodies (HtzGFP-19F7 and HtzGFP-19C8; Memorial Sloan Kettering Center, New York, NY) bound to Streptavidin MyOne T1 Dynabeads (Thermo Fisher Scientific). Beads were washed four times in a high-salt buffer containing DTT and cycloheximide. Absolutely RNA Nanoprep Kit (Agilent) was used to isolate RNA from dissected white matter samples, and RNeasy Lipid Tissue Mini Kit (QIAGEN) was used to isolate RNA from whole-brain homogenates at various circadian time points.

### RNA-sequencing

RNA recovered from TRAP was sequenced and has been uploaded to the Sequence Read Archive (National Center for Biotechnology Information; Accession code, PRJNA597018; Forbes et al., 2020). Briefly, sequencing libraries were prepared using the Trio RNA-Seq Library Preparation Kit (NuGen) for all samples with a RNA Integrity Number value > 7 (Bioanalyzer Pico Kit, Agilent). Paired-end reads (50 bp) were obtained using the NovaSeq 6000 System (Illumina), and trimmed with Trimmomatic (Bolger et al., 2014) according to the Library Preparation Kit protocol (NuGen). Reads were mapped to the mouse reference genome (Genome Reference Consortium Mouse Build 38, mm10) using STAR (Dobin et al., 2013), and read counts were generated using Htseq (Anders et al., 2015). Normalized read counts were generated using DESeq2 package for R (Love et al., 2014).

### qPCR

After TRAP isolation, RNAs were subjected to cDNA synthesis via the iScript Reverse Transcription Supermix for real-time qPCR (BIO-RAD). qPCR was performed on a CFX96 real-time system (BIO-RAD). Commercially available PrimePCR SYBR Green Assays (BIO-RAD) with proprietary sequences for the following genes were used exactly according to their recommended protocols: Arntl (UniqueAssayID: qMmuCED004960), Per1 (qMmuCED0045068), and Nr1d2 (qMmuCED0046959). Individual samples were normalized to 18S. The circadian variation evaluated via ANOVA and goodness-of-fit testing of phase-shifted sine waves.

### RNAscope

We performed RNAscope *in situ* hybridization via the manufacturer protocol (Multiplex Fluorescent Reagent Kit version 2, Advanced Cell Diagnostics) with minimal variation. Briefly, fixed, frozen brains were embedded in O.C.T. compound and cryostat sectioned into 15 μm slices. Samples were washed and then dried at 60°C before undergoing serial ethanol dehydration, a hydrogen peroxide treatment, and a protease treatment. Commercially available (Advanced Cell Diagnostics) probes for *Cspg4* (catalog #404131), *Bmal1* (catalog #438741), and *Per2* (catalog #454521) were used, followed by amplification with the manufacturer recommended fluorophores. Slides were then counterstained with DAPI before proceeding to coverslip mounting. For samples that were collected post-TBI, slides underwent a slide-mounted immunofluorescence staining protocol at the antibody concentrations indicated below for the free-floating process before proceeding with DAPI counterstaining and coverslip mounting.

### Free-floating immunofluorescence staining

Fixed, frozen brains were cryostat sectioned into 30 μm slices and stored in 30%:30% ethylene glycol/glycerol in a 1× PBS cryoprotectant solution. For staining, slices were rinsed, blocked [20% normal donkey serum (NDS), 1% bovine serum albumin (BSA), 0.3% Triton X-100], and incubated in primary antibody diluent (1% NDS, 1% BSA, 0.3% Triton X-100) with the following antibodies, as described: OLIG2 (clone 211F1.1; 1:200; catalog #MABN50, EMD Millipore); NG2 (1:250; catalog #AB5320, EMD Millipore); Ki67 (clone SolA15; catalog #14–5698-82, Thermo Fisher Scientific); GFP (1:250; catalog #GFP-1020, Aves Labs); and BMAL1 (1:250; catalog #NB100-2288, Novus Biologicals). Samples were then rinsed and incubated in donkey secondary antibodies (Jax) and coverslip mounted with ProLong Glass antifade mountant with NucBlue (Thermo Fisher Scientific).

### Image acquisition

RNA *in situ* hybridization and immunofluorescence images were acquired on a point scanning confocal microscope (model FV1000, Olympus). It was equipped with visible light (405, 442, 488, 515, and 647 nm) and pulsed infrared (MaiTai HP-OL, Spectra-Physics) lasers. It is equipped with one regular photon-multiplier tube (PMT), two spectral PMTs, two GaAsP detectors, two nondescanned detectors, and a motorized stage.

High-powered fields at 20×, 40×, and 60× objectives were used for the experiments to provide optimal resolution for the cells or features being identified: 60–100× for RNA-scope and BMAL1 quantification; and 20–40× for NG2-glia quantification. Regions of interest (ROIs) in naive animals were imaged from primary somatosensory cortex just lateral to the cingulum. For TBI experiments with cell quantification, two 40× objective high-power field were collected per slice, spanning a total area of 317 × 635 μm, centered around a peri-injury ROI at the lesion core as indicated by a combination of the following features: obvious surface deformation, increased background green fluorescence, overall cellular density, disorganized NG2 staining, and densest Ki67^+^ cell population.

### Image quantification and statistical analysis

Fiji, an open source version of ImageJ (NIH), was used for image analysis. All histologic quantifications were performed in a blinded manner. For quantification of NG2-glia at different circadian times of day, samples were collected from *Pdgfra*-TRAP animals. NG2-glia were identified as ramified cells coexpressing GFP (via the GFP-tagged ribosomal protein expression of the *Pdgfra*-TRAP animal) and NG2. Sampling our data found that ∼95.0% of GFP^+^ cells coexpressed NG2 and/or a downlineage oligodendrocyte marker (e.g., CC1), while 95.5% of GFP^+^ cells with ramified morphology coexpress NG2. For CKO experiments, NG2-glia were identified as ramified cells coexpressing NG2 and OLIG2, which are oligodendrocyte precursor cells by definition. Identification of proliferative NG2-glia required identification of ramified NG2^+^ cells expressing Ki67. All statistical testing for cell count comparisons was completed with Mann–Whitney testing.

For RNA *in situ* hybridization quantification in the naive animals, NG2-glia nuclei were identified by expression of three or more *Cspg4* punctae in DAPI-counterstained nuclei. In post-TBI experiments, NG2-glia were identified by colabeling of three or more *Cspg4* punctae with GFP immunofluorescence, as studies were performed in *Pdgfra*-TRAP animals expressing GFP-tagged ribosomes. Of note, sampling of our data found that ∼91.8% NG2-glia identified with three or more *Cspg4* punctae in uninjured cortex are associated with *Pdgfra*-TRAP expression. After NG2-glia with distinct nuclei were identified, *Bmal1* or *Per2* punctae were counted in the entire three-dimensional volume and tallied via the Fiji “Cell Counter” plugin. Data were compared through use of the Kolmogorov–Smirnov test.

For BMAL1 quantification, samples were collected from C57BL/6 mice. The signal from the DAPI channel was used to identify all nuclei on each image of a stack as independent ROIs, which were then transposed to the BMAL1 channel for intensity measurements. For each NG2-glia (i.e., Pdgfra^+^ Olig2^+^), we recorded the highest-intensity density of an ROI within the *z*-axis of a single nucleus (i.e., “peak nuclear fluorescence”) as well as the sum of intensity densities for the entire nucleus (i.e., “total nuclear fluorescence”). All intensity measurements were corrected for adjacent background fluorescence and verified to not exceed the lower and upper limits of detection during acquisition.

Data were analyzed with GraphPad Prism with statistical testing as described, unless otherwise described (Table 1). To evaluate for circadian rhythmicity of RNA, protein expression, and cell counts, we used rhythmicity analysis incorporating nonparametric (RAIN) methods. Freely available as an R/Bioconductor package, this algorithm has been validated to identify symmetric and nonsymmetric circadian rhythms (Thaben and Westermark, 2014).

**Table 1:** Statistical table

	Data Structure	Type of test	Power (Conf. Int)
a	Non-normal distribution	Rank test for umbrella alternatives (i.e., RAIN)	n/a
b	Nonlinear regression (sine)	Goodness of fit	n/a
c	Non-normal distribution	Kolmogorov–Smirnov	n/a
d	Non-normal distribution	RAIN	n/a
e	Nonlinear regression (sine)	Goodness of fit	n/a
f	Non-normal distribution	RAIN	n/a
g	Nonlinear regression (sine)	Goodness of fit	n/a
h	Non-normal distribution	Mann–Whitney	Rank sum: 36 vs 19, *U* = 4
i	Non-normal distribution	Mann–Whitney	Rank sum: 11 vs 10, *U* = 4
j	Non-normal distribution	Mann–Whitney	Rank sum: 8 vs 13, *U* = 2
k	Non-normal distribution	Mann–Whitney	Rank sum: 44 vs 11, *U* = 1
l	Non-normal distribution	Mann–Whitney	Rank sum: 43 vs 12, *U* = 2
m	Non-normal distribution	Mann–Whitney	Rank sum: 35 vs 10, *U* = 0
n	Non-normal distribution	Mann–Whitney	Rank sum: 30 vs 15, *U* = 0
o	Non-normal distribution	Mann–Whitney	Rank sum: 30 vs 15, *U* = 0

## Results

### NG2-glia express clock genes, including *Bmal1*, that oscillate in a circadian manner

**Figure 1.**
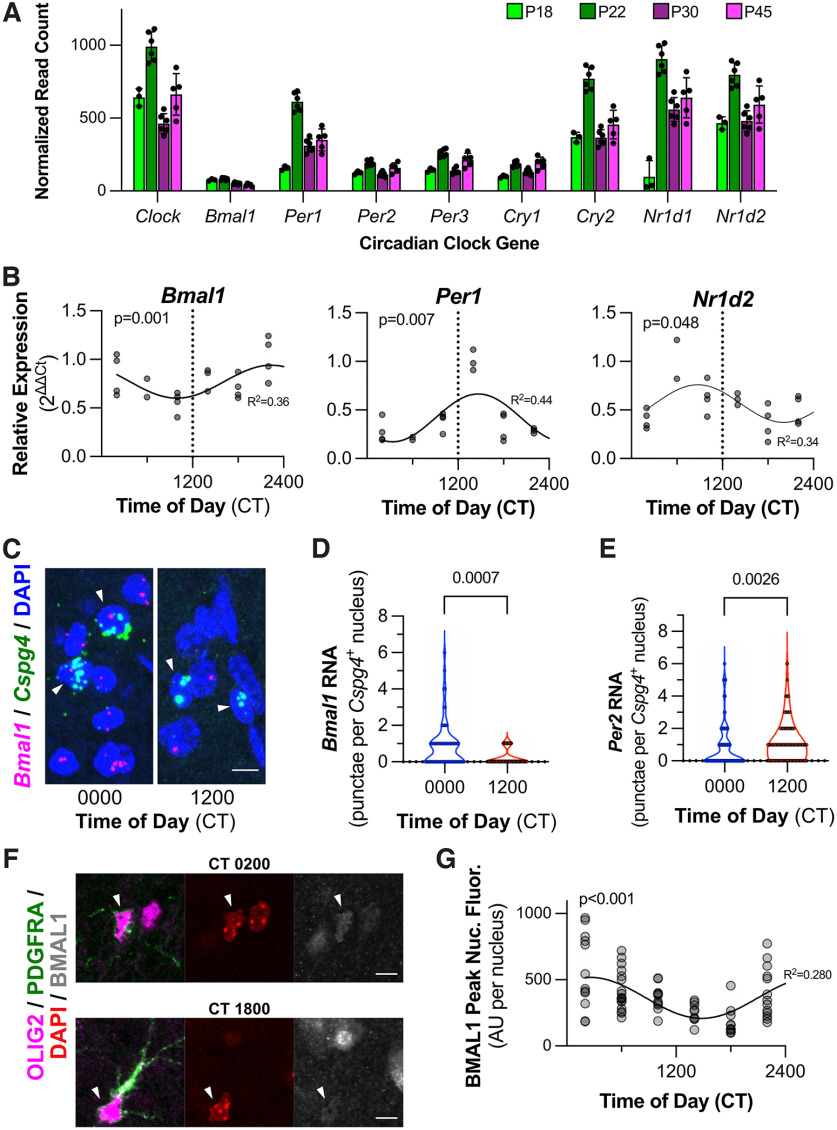
Circadian clock gene expression, including *Bmal1*, oscillate in NG2-glia. ***A***, Bar graph depicting normalized read counts of circadian clock gene transcripts isolated via TRAP-seq from *Pdgfra*-TRAP mice. Samples were collected at P18, P22, P30, and P45. Each dot is representative of one animal. (mean ± SD). ***B***, Graphs depicting relative expression, via qPCR, of circadian clock gene transcripts (*Bmal1*, left; *Per1*, middle; *Nr1d2*, right) isolated from *Pdgfra*-TRAP mice throughout several times of day (CT 0200, 0600, 1000, 1400, 1800, and 2200). Each dot is representative of one animal (*n* = 21 over six groups). The *p*-values from RAIN^a^. *R*^2^ is from goodness of fit to depicted sine wave for illustrative purpose^b^. ***C***, Representative images of RNA ISH of *Bmal1* (magenta) and *Cspg4* (green) with nuclear staining (DAPI, blue) at CT 0000 and 1200 in somatosensory cortex. *Cspg4*^+^ cells are denoted with solid arrowheads (▴). Scale bar, 10 μm. ***D***, Graph depicting quantification of *Bmal1* RNA ISH in cortical *Cspg4*^+^ cells at CT 0000 and CT 1200 in somatosensory cortex. *N* = 3 animals/condition (68 and 77 cells, respectively). The *p*-value is from the Kolmogorov–Smirnov test^c^. ***E***, Graph depicting quantification of *Per2* RNA ISH in cortical *Cspg4*^+^ cells at CT 0000 and CT 1200 in somatosensory cortex. *N* = 3 animals/condition (134 and 81 cells, respectively). The *p*-value is from the Kolmogorov–Smirnov test^c^. ***F***, Representative images of immunofluorescence for nuclear BMAL1 (gray) in OLIG2^+^ (magenta) PDGFRA^+^ (green) NG2-glia in somatosensory cortex. Nuclei are stained with DAPI (red). NG2-glia nuclei are indicated with solid arrowheads (▴). Scale bar, 10 μm. ***G***, Graph depicting quantification of peak intensity of nuclear BMAL1 immunofluorescence in *Pdgfra*-expressing NG2-glia from somatosensory cortex at several times of day (CT 0200, 0600, 1000, 1400, 1800, and 2200). *N* = 2–3 animals/time point (73 nuclei total). Each dot is representative of one nucleus. The *p*-value is from RAIN^d^. *R*^2^ is from the goodness-of-fit to depicted sine wave for illustrative purpose^e^.

The mammalian molecular clock is characterized by the 24 h oscillation of several component clock genes, including the core loop transcriptional activators (*Bmal1*, *Clock*) and inhibitors (*Per1*, *Per2*, *Per3*, *Cry1*, *Cry2*), and secondary loop components (e.g., *Nr1d1*, *Nr1d2*; Takahashi, 2017). We first set out to determine whether NG2-glia possess detectable levels of these circadian clock gene transcripts. Using TRAP, we isolated ribosome-bound mRNAs specifically from NG2-glia by using *Pdgfra*-TRAP mice (Forbes et al., 2020). Through our sequencing of NG2-glia mRNAs, we found detectable and consistent quantities of all the aforementioned clock genes at multiple developmental time points, ranging from early in postnatal development (P18) to young adulthood (P45; Fig. 1*A*). We then used quantitative PCR to characterize the 24 h oscillation of translation of specific clock gene transcripts from NG2-glia, finding rhythmic expression of clock genes from the core (*Bmal1*, *Per1*) and secondary (*Nr1d2*) loops (Fig. 1*B*). Consistent with clock gene timing seen in other tissues (Takahashi, 2017), *Bmal1* translation appears to reach a peak and nadir around circadian time (CT) 2200 and CT 1000, respectively. Conversely, *Per1* translation is out of phase with *Bmal1*, with a peak and nadir at CT 1400 and between CT 0200 and 0600, respectively, and is also consistent with the timing seen in other tissues.

10.1523/ENEURO.0110-22.2022.f1-1 Figure 1-1 Circadian variation in total nuclear BMAL1 expression. Graph depicting quantification of total intensity of nuclear BMAL1 immunofluorescence in *Pdgfra*-expressing NG2-glia at several times of day (CT 0200, 0600, 1000, 1400, 1800, and 2200). *N* = 2–3 animals/time point (68 nuclei total). Each dot is representative of one nucleus. The *p*-value is from RAIN^d^. *R*^2^ is from the goodness of fit to the depicted sine wave for illustrative purpose^e^. Graph depicting quantification of total intensity of nuclear BMAL1 immunofluorescence in *Pdgfra*-expressing NG2-glia at several times of day (CT 0200, 0600, 1000, 1400, 1800, and 2200). n/a, Not applicable. Download Figure 1-1, file.

We then sought to confirm our TRAP findings with small-molecule fluorescent RNA ISH (RNAscope). We initially focused on *Bmal1* expression as it is an essential, nonredundant component of the mammalian circadian clock (Haque et al., 2019) and is an integral regulator of circadian physiology in several cell types (Lefta et al., 2012; Jiang et al., 2021). Cortical NG2-glia, identified by *Cspg4*^+^ nuclei, demonstrated increased *Bmal1* RNA expression at a circadian time of day near the expected peak of *Bmal1* expression (CT 0000) versus the daily nadir (CT 1200; Fig. 1*C*,*D*). To ensure that this was not because of a global change in NG2-glia RNA expression between the two time points, we also evaluated cortical NG2-glial *Per2* expression, revealing increased *Per2* RNA levels at CT 1200 relative to CT 0000 (Fig. 1*E*), also as expected for those times of day (Takahashi, 2017).

Given the cycling of *Bmal1* RNA in cortical NG2-glia, our next step was to determine whether BMAL1 protein expression also oscillates in a circadian manner. By quantifying nuclear immunofluorescence intensities throughout the 24 h day, we found that BMAL1 is rhythmically expressed with a peak and trough at CT 0200 and CT 1400–1800, respectively [Fig. 1*F*,*G*, Extended Data Fig. 1-1; peak nuclear fluorescence: *n* = 2–3 animals (9–18 nuclei) per time point, *p* < 0.001^d^; total nuclear fluorescence: *n* = 2–3 animals (9–15 nuclei) per time point, *p* < 0.001^e^]. The peak and trough of BMAL1 protein expression was narrowly phase shifted following the pattern of *Bmal1* RNA expression (Fig. 1*B*), which is consistent with the ability of TRAP to isolate actively translating RNAs. Thus, we posit that cortical NG2-glia exhibit rhythmic expression of circadian clock genes, including *Bmal1*, suggesting that BMAL1-regulated transcriptional programs are subject to circadian control.

### Endogenous *Bmal1* expression regulates cortical NG2-glia proliferation in adult, naive cortex

**Figure 2.**
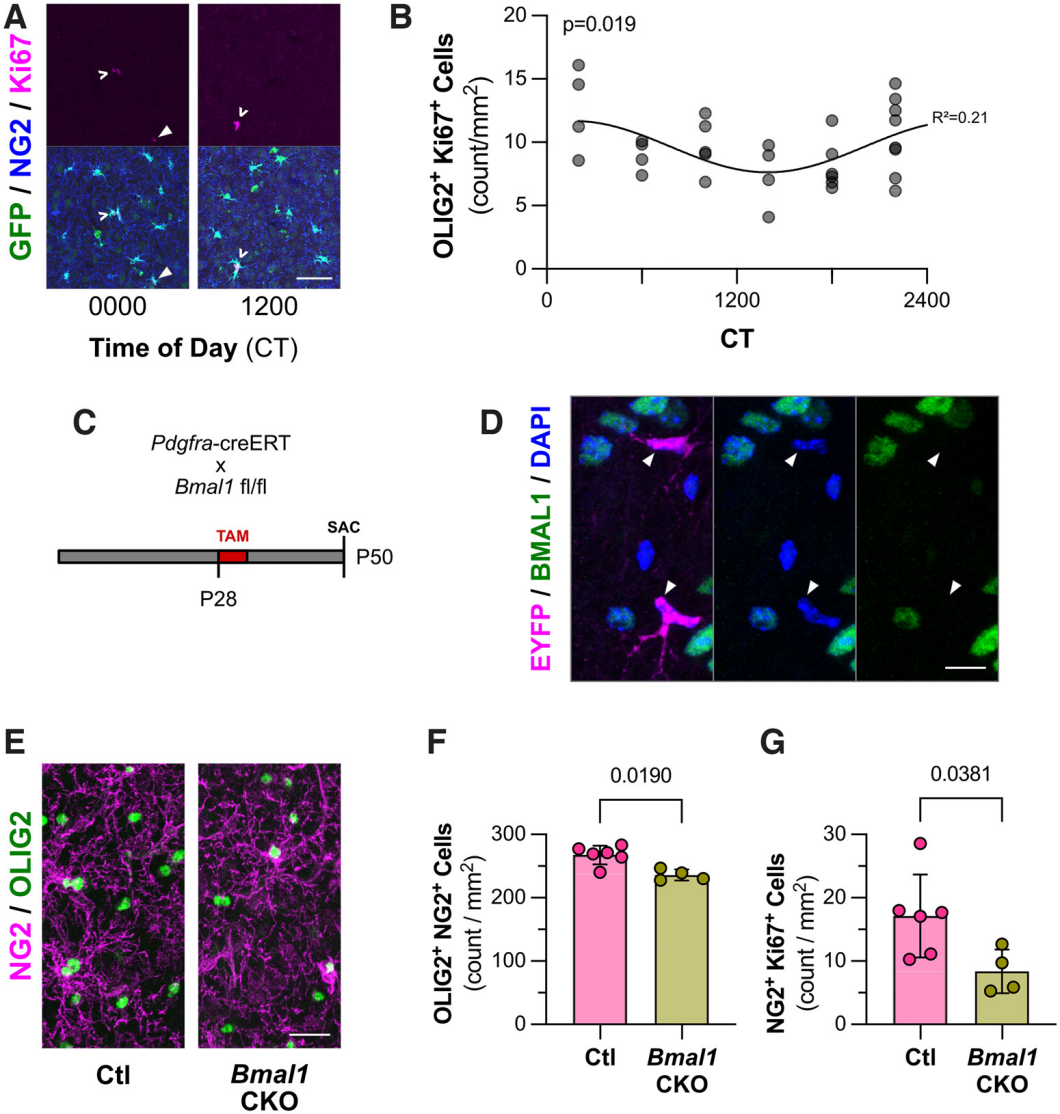
Circadian oscillation of *Bmal1* regulates NG2-glia proliferation in naive cortex. ***A***, Representative images of immunofluorescence for cortical NG2-glia from somatosensory cortex at CT 0000 and CT 1200. NG2-glia from *Pdgfra*-TRAP mice identified as ramified NG2^+^ GFP^+^ cells, with proliferative cells indicated by Ki67 staining (magenta). Proliferative single NG2-glia are indicated by a solid arrowhead (▴), and doublets are indicated by a caret (^). Scale bar, 50 μm. ***B***, Graph depicting quantification of OLIG2^+^ Ki67^+^ density in somatosensory cortex of C57BL/6 mice at several times of day (CT 0200, 0600, 1000, 1400, 1800, and 2200). *N* = 4–8 animals/time point (31 total). Each dot is representative of one animal. The *p*-value is from RAIN^f^. *R*^2^ is from the goodness of fit to depicted sine wave for illustrative purpose^g^. ***C***, Mating scheme and experimental timeline for *Bmal1* CKO experiments. ***D***, Representative images of immunofluorescence for BMAL1 in CKO mice (*Pdgfra*-creERT:*Bmal1*^fl/fl^:R26R-EYFP), indicated by colabeling for EYFP reporter (magenta), DAPI (blue), and BMAL1 (green). Nuclei from recombined NG2-glia with EYFP reporter, but lacking BMAL1 staining, are indicated by a solid arrowhead (▴). Scale bar, 10 μm. ***E***, Representative images of immunofluorescence for NG2-glia, indicated by colabeling for NG2 (magenta) and OLIG2 (green) from cortex of *Pdgfra*-creERT:*Bmal1*^fl/fl^ CKO and Ctl *Pdgfra*-creERT:*Bmal1*^+/+^ mice 3 weeks following tamoxifen injection. Samples collected at CT 0000. Scale bar, 20 μm. ***F***, Graph depicting quantification of NG2-glia (NG2^+^ and OLIG2^+^) from cortices of CKO and Ctl mice 3 weeks following tamoxifen injection. Samples were collected at CT 0000. Each dot is representative of one animal (mean ± SD). The *p*-value is from Mann-Whitney test^k^. ***G***, Graph depicting quantification of proliferative NG2^+^ Ki67^+^ cell density from cortices of CKO and Ctl mice 3 weeks following tamoxifen injection. Samples collected at CT 0000. Each dot is representative of one animal (mean ± SD). The *p*-value is from Mann-Whitney test^l^.

NG2-glia appear to have the fastest basal rate of proliferation (∼1.5% of the population) among the resident cell types of the adult cortex, as far fewer cortical NG2-glia undergo differentiation into mature oligodendrocytes, as typically seen in earlier stages of development (Zhu et al., 2011; Hughes et al., 2013; Forbes et al., 2020). Thus, we chose to evaluate the role that circadian rhythm may play in adult cortical NG2-glia proliferation. Via immunofluorescence of samples collected at multiple times throughout the circadian day, we found that NG2-glia demonstrate a circadian rhythmicity of proliferation, as indicated by strong Ki67^+^ expression (Fig. 2*A*,*B*), suggesting that cell proliferation is coordinated with time of day. Interestingly, the peak and trough of Ki67 expression exhibited a similar circadian pattern as *Bmal1* expression (Fig. 1*B*,*G*). However, when comparing the total NG2-glia densities at times of day in which *Bmal1* expression and NG2-glia proliferation are at their highest and lowest, we found only a nonsignificant trend toward a higher density at CT 0000 (254.6 ± 13.9 vs 237.9 ± 14.2 cells/mm^2^, *n* = 5 mice/condition, *p* = 0.0952^h^). The small degree of difference was unsurprising given the tight homeostatic control of NG2-glia density demonstrated in previous *in vivo* imaging studies (Hughes et al., 2013).

We tested the correlation between *Bmal1* expression and NG2-glia cell proliferation through a tamoxifen-inducible CKO of *Bmal1* restricted to NG2-glia via the *Pdgfra* promoter (Fig. 2*C*). In our hands, 5 d of tamoxifen administration in *Pdgfra*-creERT::Bmal1(fl/fl)::R26R-EYFP reporter mice resulted in ∼95.3% recombination efficiency (i.e., EYFP^+^ NG2^+^ OLIG2^+^ vs all NG2^+^ OLIG2^+^ cells). Furthermore, we found that that this approach indeed knocked out BMAL1 protein expression in recombined cells (Fig. 2*D*), without causing any significant changes in sleep activity during light or dark phases [light phase: CKO = 2.97 ± 5.04% (mean ± SD), Ctl = 4.77 ± 6.89%, *p* > 0.99^i^; dark phase: CKO = 11.78 ± 6.02%, Ctl = 6.05 ± 2.93%, *p* = 0.40^j^; *n* = 3/group]. Following *Bmal1* knockdown at P28, mice at P50 demonstrated a significant reduction at CT 0000 in total NG2-glia cell density (Fig. 2*E*,*F*; 267.5 ± 14.9 vs 235.9 ± 8.8 cells/mm^2^, *n* = 4–6 mice/condition, *p* = 0.0190^k^) and NG2^+^ cells proliferation (Fig. 2*G*; 17.1 ± 6.6 vs 8.4 ± 3.5 cells/mm^2^, *n* = 4–6 mice/condition, *p* = 0.0381^l^). A similar decrease in proliferative Ki67^+^ OLIG2^+^ cells was also seen at CT 1200 (18.8 ± 4.0 vs 10.0 ± 3.9 cells/mm^2^, *n* = 4–5 mice/condition, *p* = 0.0159^m^). These data are consistent with a role for BMAL1 in maintaining a basal cell density in the naive, uninjured cortex through the regulation of proliferation.

### Endogenous *Bmal1* expression regulates NG2-glia proliferation after TBI

**Figure 3.**
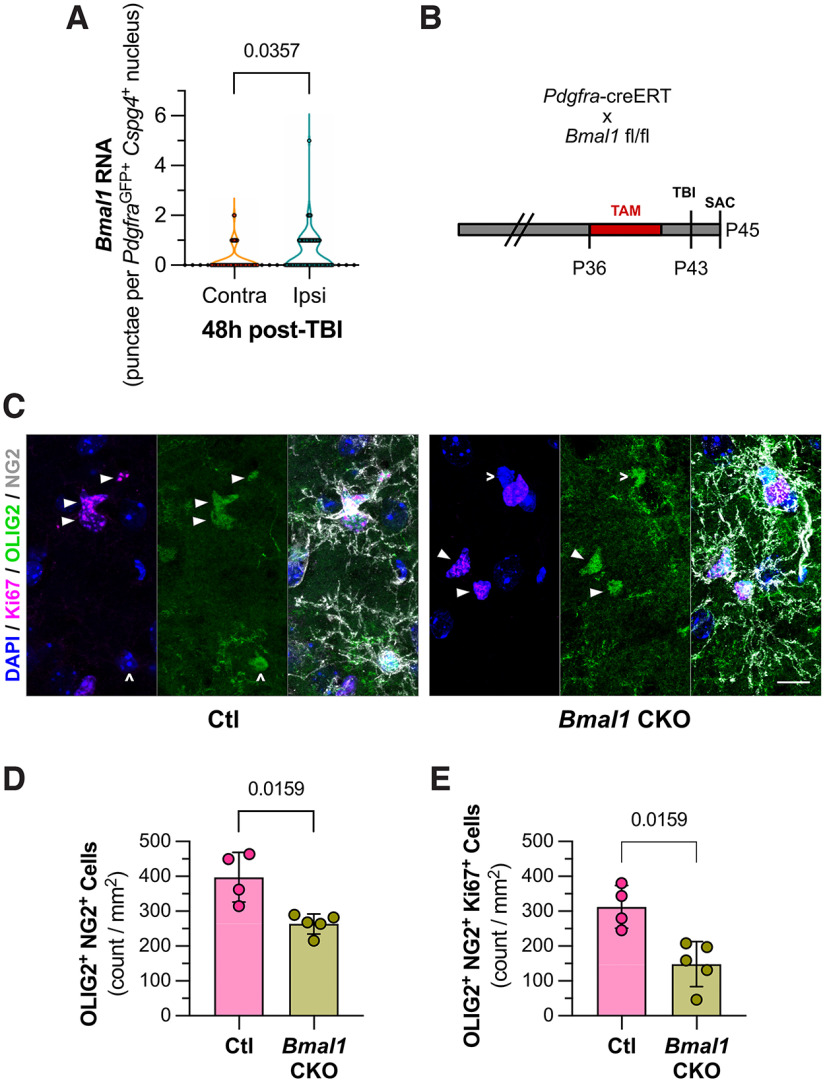
Endogenous *Bmal1* expression regulates NG2-glia proliferation after TB1. ***A***, Graph depicting quantification of *Bmal1* RNA ISH in *Ng2*^+^ *Pdgfra*^TRAP-GFP^ cells from cortex adjacent (Ipsi) and contralateral (Contra) to the injury. Samples collected at CT 1200. *N* = 4 animals. The *p*-value from Kolmogorov–Smirnov test^c^. ***B***, Mating scheme and experimental timeline for *Bmal1* CKO TBI experiments. ***C***, Representative images of immunofluorescence for proliferative NG2-glia in cortical penumbra at 48 h after TBI from *Pdgfra*^creERT^*Bmal1*^fl/fl^ CKO and pooled Ctl animals. Proliferative NG2-glia [NG2^+^ (gray) OLIG2^+^ (green) Ki67^+^ (magenta)] are indicated with solid arrowheads (▴), while nonproliferative NG2-glia (NG2^+^ OLIG2^+^ Ki67^–^) are indicated by caret (^). DAPI nuclear staining in blue. Scale bar, 10 μm. ***D***, Graph depicting quantification of NG2-glia (NG2^+^ OLIG2^+^) in cortical penumbra at 48 h after TBI from CKO and Ctl mice. Each dot is representative of one animal (mean ± SD). The *p*-value is from Mann-Whitney test^n^. ***E***, Graph depicting quantification of proliferative NG2-glia (NG2^+^ OLIG2^+^ Ki67^+^) in cortical penumbra at 48 h after TBI from CKO and Ctl mice. *N* = 5 and 4, respectively. Each dot is representative of one animal (mean ± SD). The *p*-value is from Mann-Whitney test^o^.

Given the link between *Bmal1* expression and NG2-glia density and proliferation in the naive, uninjured animal, we next sought to determine whether *Bmal1* played a role in the proliferative response to injury as well. We hypothesized that *Bmal1* expression was necessary for NG2-glia proliferation after an acute brain insult like TBI. Indeed, via RNAscope, we found that within the same animals at 48 h after TBI, injury-adjacent NG2-glia exhibited upregulation of *Bmal1* RNA versus those in contralateral, uninjured cortices (Fig. 3*A*). Of note, these levels are comparable to the peak and trough of *Bmal1* expression seen at CT 0000 and CT 1200, respectively (Fig. 1*D*). We next performed TBI in our *Bmal1* CKO mice (Fig. 3*B*), finding a reduction in the postinjury NG2-glia density in the lesion core (Fig. 3*C*,*D*; 397.4 ± 71.5 vs 263.4 ± 29.0 cells/mm^2^, *n* = 4–5 mice/condition, *p* = 0.0159^n^), remaining at levels similar to those seen in uninjured animals (Fig. 2*A*,*D*). Furthermore, while TBI induces an increase in proliferative Ki67^+^ NG2-glia by an order of magnitude (compare Figs. 3*E*, 2*G*), this population was also reduced by *Bmal1* CKO (Fig. 3*E*; 312.4 ± 61.0 vs 148.3 ± 64.8 cells/mm^2^, *n* = 4–5 mice/condition, *p* = 0.0159°). These data suggest that *Bmal1* expression regulates the NG2-glia proliferative response to TBI.

## Discussion

This is the first description of the presence and oscillation of circadian clock genes specifically in NG2-glia. While NG2-glia do receive inputs from neurons (Li et al., 2020), which may play a role in synchronizing the endogenous clock with the master clock, we show here that a component of the endogenous clock, *Bmal1*, regulates a key cellular function, proliferation. These data are consistent, with the clock serving as part of a cell-autonomous mechanism to regulate basal proliferation that is distinct from neuronal inputs that direct NG2-glial cellular functions (Li et al., 2020). Furthermore, as the circadian clock is composed of transcription factors [e.g., BMAL1/CLOCK transcriptional activator, RAR-related orphan receptor (ROR), and REV-ERB] with widespread response elements (e.g., E-box *cis*-regulatory elements, ROR response elements; Takahashi, 2017), we posit that several other NG2-glia processes are also under control of the endogenous clock. Characterization of clock gene targets will significantly advance our understanding of the functional roles of NG2-glia in the brain throughout the 24 h day. Additionally, as we focused primarily on cortical NG2-glia in adulthood, it will be critically important to investigate how the circadian regulation of NG2-glia may be subject to other regulatory mechanisms, including regional heterogeneity (Dimou et al., 2008) and developmental age (Yang et al., 2016).

In addition to their functions in the resting brain, NG2-glia play an important role in ameliorating acute and chronic insults throughout the CNS (Dimou and Gallo, 2015; Adams and Gallo, 2018; Psenicka et al., 2021). Interestingly, while *Bmal1* expression does cycle throughout the circadian day in the naive brain, it is also induced UPON CNS injury. In both cases, NG2-glia proliferation appears to be dependent on *Bmal1* expression, raising the possibility that this transcription factor serves in a common pathway to engage molecular programs for proliferation. Consequently, the circadian dependence of the NG2-glia regenerative potential has significant implications for the treatment of CNS injury and disease. Because neurologic disorders frequently induce circadian rhythm disturbances (Logan and McClung, 2019), it is possible that these circadian dysrhythmias, themselves, play a role in impairing cellular recovery at the molecular level in NG2-glia. These findings underscore the importance of addressing circadian dysregulation in neurologic diseases to maximize brain healing.

## Synthesis

Reviewing Editor: Silvia Pagliardini, University of Alberta Decisions are customarily a result of the Reviewing Editor and the peer reviewers coming together and discussing their recommendations until a consensus is reached. When revisions are invited, a fact-based synthesis statement explaining their decision and outlining what is needed to prepare a revision will be listed below. The following reviewer(s) agreed to reveal their identity: Christopher Colwell.

The role of the circadian clock in regulating the brain’s regenerative potential has been understudied. In this manuscript, the authors examine murine NG2-glia which is a large population of proliferative cells in the mature CNS. They report that these cells rhythmically express circadian clock genes in a 24-hour period (Bmal1 and Per1 mRNA). They show that the peak of Bmal1 expression is also when there is a peak of daily NG2-glia proliferation. To explore this correlation, the authors examine the impact of a conditional knockout (CKO) of Bmal1 and find that the KO decreases both cortical NG2-glia density and cellular proliferation. Furthermore, in a neurotrauma model, they show that pathology-induced NG2-glia is also compromised in the Bmal1 CKO.

The methods, which include TRAP-qPCR, RNAscope, and tissue-specific deletion of BMal1 in NG2 cells using Pdgfra-CreERT, are very strong, and the data are clear and straightforward. The findings provide important initial data that circadian clock genes may regulate NG2 cell function.

There are a number of strengths to this work.

The role of the circadian clockwork in the NG2-glia cell population is an important and unexplored issue.

The use of the Pdgfra-TRAP is powerful and the use of the conditional, targeted Bmal1 KO is very appropriate.

The cellular imaging is of high quality.

Still there are some important concerns that need to be addressed.

First, throughout the text, the authors need to clarify the brain region and the age of the tissue shown as it was difficult to find this information. Maybe just adding it to the figure legends would be sufficient.

Second, the experiments are framed as circadian but for most of the experiments the measurements at made at CT 0 and 12. We do not know if these are the peak and trough of the protein expression. This is never shown and it is just not possible to show the rhythm in Bmal expression and to identify the peak and trough of protein levels. It is not even day and night but phases projected from the prior LD cycle and collected when the mice are in DD. I guess that we are looking at a projected day/night difference.

The circadian rhythm analysis of transcripts in Fig. 1 uses cosinor analysis, but a more rigorous method such as JTK_cycle/Metcycle would be more convincing.

More expansion of the characterization of the cKO mouse is needed. Specifically, in Fig. 2E, why is there eGFP? Is this expressed alongside Cre? How was the recombination calculated. Do these mice have any overt behavioral rhythm changes (wheel running, etc)?

The authors make the claim that there is a correlation between Bmal1 expression and proliferation. This would be an important test of their hypothesis but was not examined. They would need to make measurements of BMAL1 and Ki67+ in the 6 time points to really look at the rhythm and see if the peaks and troughs alien.

In the Bmal KO, they show a decrease in proliferation as measured by Ki67. But at what time of the cycle? Is the difference in projected day and night (CT 0 and 12) lost in the KO line?

In Fig. 2 F, G, is there any indication of NG2 cell death caused by Bmal1 deletion?

In the TBI model, they show an increase in Bmal after the TBI. Does proliferation as measured by Ki67 also increase? This would support their hypothesis. In the figure showing the results of this experiment do they have untreated controls? I think that all of the mice received the TBI but this should be clarified.

In Fig. 3, is there some overall measure of the degree of injury caused in control and cKO mice by TBI? It could be that the degree of damage is much worse in one or the other and this influences NG2 proliferation.

## Author Response

RESPONSES FOR REVIEWERS:

We thank the reviewers and eNeuro editors for the opportunity to consider these points.

The reviewer’s comments will be set apart by double-asterisks (**), and our responses follow. Please forgive the lack of additional formatting (e.g. color, bolding, italics) which is not permissible according to resubmission guidelines.

**First, throughout the text, the authors need to clarify the brain region and the age of the tissue shown as it was difficult to find this information. Maybe just adding it to the figure legends would be sufficient. **

Thank you for this point. We have simplified the Methods (pages 8-9) and addended the Figure Legends (e.g. Fig 1C-G).

**Second, the experiments are framed as circadian but for most of the experiments the measurements at made at CT 0 and 12. We do not know if these are the peak and trough of the protein expression. This is never shown and it is just not possible to show the rhythm in Bmal expression and to identify the peak and trough of protein levels. It is not even day and night but phases projected from the prior LD cycle and collected when the mice are in DD. I guess that we are looking at a projected day/night difference. **

Thank you for this point. As requested below, we now identify the peak and trough of BMAL1 protein expression throughout the 24-hr day (See below for details).

**The circadian rhythm analysis of transcripts in Fig. 1 uses cosinor analysis, but a more rigorous method such as JTK_cycle/Metcycle would be more convincing. **

Thank you for this suggestion. In reviewing the available algorithms, we wanted to ensure the detection of both sinusoidal (e.g. cosine) and non-symmetric (e.g. sawtooth) circadian rhythmicity, especially given the non-symmetric manner in which Bmal1 oscillates in liver (Chudhova et al. 2009 Bioinformatics). We utilized Rhythmicity Analysis Incorporating Non-parametric methods (RAIN), a validated and freely available package for R/Bioconductor (Thaben and Westermark, 2014 J Biol Rhythms). We have addended the Methods to include this algorithm (page 11) and applied it to analyze circadian rhythmicity of various data throughout the paper (i.e. qPCR data, immunofluorescence data) with a 24hr period. The resultant p-values are now listed within figures (e.g. Fig 1B, Fig 1G), and we have updated the figure legends, and added the relevant reference. As this analysis does not provide a curvefitting output, we do also continue to provide the fit sine wave provided by Graphpad with a goodness of fit statistic (R2 value) for illustrative purposes and specify this in the figure legends as well so readers do not confuse the statistical methods utilized.

**More expansion of the characterization of the cKO mouse is needed. Specifically, in Fig. 2E, why is there eGFP? Is this expressed alongside Cre? How was the recombination calculated. **

Thank you for pointing out the confusion in Fig 2E (currently listed as Fig 2D in the revised version submitted here). For that panel, we combined the Bmal1 CKO strategy (Pdgfra-creERT::Bmal1(fl/fl) with a R26R-EYFP transgenic mouse line that expresses the EYFP reporter in cells in which Cre recombinase was induced by tamoxifen (Pdgfra-creERT::R26R-EYFP::Bmal(fl/fl)). While mention of the R626R-EYFP Jax stock was mentioned in the methods, a description of its use was indeed lacking. By using the Rosa EYFP reporter, we can calculate recombination by comparing the number of EYFP+ cells with the number of total cells expressing NG2-glia markers (NG2 and OLIG2). We have now included mention of this process it in the Results (page 14) and updated the figure legend.

**Do these [CKO] mice have any overt behavioral rhythm changes (wheel running, etc)? **

Thank you for this question. We now provide sleep-wake data using a non-invasive piezoelectric sleep recording system. These data suggest that CKO does not produce a significant difference in the daily distribution of motor activity or sleep (as the system relies on movement for sleep detection) in the light and dark periods. We have added these data to the Results (page 14), addended the Methods (page 6), and included two references.

**The authors make the claim that there is a correlation between Bmal1 expression and proliferation. This would be an important test of their hypothesis but was not examined. They would need to make measurements of BMAL1 and Ki67+ in the 6 time points to really look at the rhythm and see if the peaks and troughs align. **

Thank you for this comment - this was mentioned in part by the previous reviewer as well. We now provide a 24-hour series of BMAL1 expression (Fig 1G and Extended Fig 1.1) as well as a 24-hr series of Ki67 expression (Fig 2B). It appears that the peak of BMAL1 expression (0200) only slightly lags behind the peak of TRAP Bmal1 RNA expression (2200), which is as expected given that the TRAP technique specifically isolates actively translating RNAs, and so protein levels should be relatively tightly correlated (unlike other mRNA isolation techniques which may show peaks several hours before protein levels rise). This also corresponds with the peak of Ki67+ cell counts. The Results section has been updated to reflect this (page 13-14). We have updated the Methods section (page 11) to reflect this entirely new experimental cohort of mice and also clarified the description of the methods used to analyze BMAL1 immunofluorescence. Of note, because the 24-hr time series did require more space within Figure 2, we did have to move the previous Figure 2B, which was a single bar graph, to the Results text - it was a non-statistically-significant result, anyhow).

**In the Bmal KO, they show a decrease in proliferation as measured by Ki67. But at what time of the cycle? Is the difference in projected day and night (CT 0 and 12) lost in the KO line? **

We thank the reviewers for pointing out this confusion. The original submission included the effect of the CKO on NG2-glia proliferation at CT 0000, the time of day during which Bmal1 expression was expected to be highest. We have now repeated the CKO experiment with an entirely new cohort of transgenic mice at CT1200 and found that proliferation can also be stunted at that time of day as well. This is included in the Results (page 14).

**In Fig. 2 F, G, is there any indication of NG2 cell death caused by Bmal1 deletion? **

This was a very interesting question. We performed cleaved caspase-3 staining, which has been used extensively in the NG2-glia/OPC literature as a marker of NG2-glia apoptosis (e.g. Jablonska et al 2012 J Neurosci; Caprariello et al 2015 J Neurosci; Ming et al. 2013 J Neurosci). We found that the number of NG2-glia with activated caspase-3 staining in the naïve adult cortex was far too few to be able to perform comparisons between CKO and Ctl groups (i.e. only one CC3+ NG2-glia per several slices of entire cortex per animal), in line with the reports of very low rates of NG2-glia apoptosis seen in mature cortex (Hughes et al 2013 Nat Neurosci). Because these data cannot be relied upon to properly support or refute a role for Bmal1 in NG2-glia cell death, we did not include mention of it in the manuscript.

**In the TBI model, they show an increase in Bmal after the TBI. Does proliferation as measured by Ki67 also increase? Proliferation as measured by Ki67 does indeed increase following TBI. This would support their hypothesis. **

In Figure 3E, we do find that the number of Ki67+ NG2-glia following TBI is on the order of ∼300 cells/mm2. This is an order of magnitude greater than the number of Ki67+ NG2-glia in the naïve, uninjured brain reported in Figure 2C and 2H (∼15-30 cells/mm2). We have addended the Results to reinforce the increase in proliferative NG2-glia that are seen after injury (page 15).

**In the figure showing the results of this experiment do they have untreated controls? I think that all of the mice received the TBI but this should be clarified. **

We apologize for the lack of clarity. In an effort to provide the most rigorous control for the effect of TBI, itself, on Bmal1 expression, we provided a comparison between the injured cortex and the contralateral uninjured cortex within the same mice (n=4 animals); this allows for control for anesthetic administration and any changes in systemic vital signs that could be experienced post-injury. We have addended the text to further reinforce that the comparisons are made between the ipsilateral and contralateral sides (Results page 15). That said, we were sure to also make note that these TBI experiments were conducted at CT 1200 (Fig Legend 3A), the time of day when Bmal1 expression is near its nadir; these data are therefore comparable to the RNAscopeTM characterization of Bmal1 expression at CT 1200 in uninjured animals in Fig 1C-D. Of note, the post-injury contralateral Bmal1 expression at CT 1200 (Fig 3A) was minimal, as was the naïve brains at CT 1200 (Fig 1C-D). We have now addended the text (Results page 15) the text also reflect this.

**In Fig. 3, is there some overall measure of the degree of injury caused in control and cKO mice by TBI? It could be that the degree of damage is much worse in one or the other and this influences NG2 proliferation. **

We wanted to ensure that our comparisons between two conditions (CKO and Ctl) were between areas with similar pathological processes and cellular response. Because NG2-glia play such a key role in cellular regeneration following TBI, we chose to strictly define the lesion core as our region of interest. To ensure an apples-to-apples comparison, we used a strict total area of consideration (317-635 um) and outlined the lesion core, specifically, based on histological parameters described in the methods (i.e. obvious surface deformation, increased background green channel autofluorescence, cellular density, and disorganized NG2+ staining). By focusing on the lesion core, only, we feel that a comparison between CKO and Ctl mice will not be influenced by changes in degree of injury (e.g. lesion size) or more global phenomenon that accompany TBI. We have adjusted the text to better inform readers of our focus on the lesion core (Methods page 10, Results page 15).
